# Moisture is not always bad: H_2_O accelerates the conversion of DMAPbI_3_ intermediate to CsPbI_3_ for boosting the efficiency of carbon-based perovskite solar cells to over 16%

**DOI:** 10.1016/j.fmre.2022.07.005

**Published:** 2022-07-24

**Authors:** Hailiang Wang, Huicong Liu, Zijing Dong, Xueyuan Wei, Weiping Li, Liqun Zhu, Cheng Zhu, Yang Bai, Haining Chen

**Affiliations:** a School of Materials Science and Engineering, Beihang University, Beijing 100191, China; b Beijing Key Laboratory of Construction Tailorable Advanced Functional Materials and Green Applications, MIIT Key Laboratory for Low-dimensional Quantum Structure and Devices, Experimental Center of Advanced Materials, School of Materials Science & Engineering, Beijing Institute of Technology, Beijing 100081, China

**Keywords:** Inorganic perovskite, CsPbI_3_, Carbon electrode, Solar cells, Water

## Abstract

Inorganic CsPbI_3_ perovskite has exhibited great application potential in perovskite solar cells (PSCs) due to its suitable optical bandgap and high chemical stability. However, the perovskite phases of CsPbI_3_ are not stable at room temperature, where they transition to non-perovskite phases. Humidity or water has been thought to be the primary factor inducing this phase transition, which should be avoided throughout the procedure of film and device processing. Surprisingly, the present study indicates that preparing a precursor solution in humid air is beneficial to the growth of high-quality CsPbI_3_ perovskite to enhance device performance. It is demonstrated that the incorporation of H_2_O in the precursor solution from humid air or by intentional addition significantly changes the composition of coordination compounds and increases the amount of low iodine coordination complexes. As a result, the crystallization of dimethylammonium lead iodide (DMAPbI_3_) intermediate is suppressed well, which accelerates its subsequent conversion to CsPbI_3_ perovskite. Consequently, an oriented CsPbI_3_ perovskite film with improved crystallinity and lower defect density is obtained. Most importantly, carbon-based PSCs (C-PSCs) based on the CsPbI_3_ perovskite film achieve an efficiency of 16.05%, a new record for inorganic C-PSCs.

## Graphical abstract

**Figure:**
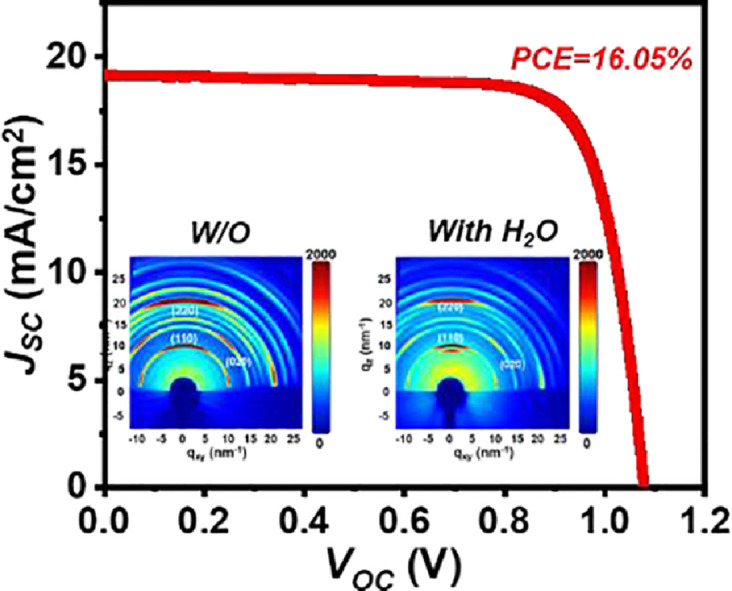

## 1 Introduction

Solution-processed organic–inorganic hybrid perovskite solar cells (PSCs) have achieved comparable power conversion efficiencies (PCEs, over 25%) with commercial Si-based cells [1,2]. However, the susceptibility of organic–inorganic hybrid perovskite to degradation in high-humidity and high-temperature environments limits its stability, which greatly inhibits the commercialization of PSCs. To solve this issue, replacing organic–inorganic hybrid perovskites with purely inorganic perovskites is a promising strategy. Thus far, great progress has been made in research on inorganic PSCs, and 21% PCEs have been achieved [3], [4], [5], [6], [7], [8], [9], [10], [11], [12], [13], [14], [15], [16]. Furthermore, by simultaneously substituting the organic hole transport layer and metal electrode with a carbon electrode, so-called C-PSCs, device stability can be further enhanced [17], [18], [19], [20], [21]. Recently, over 15% PCE has been achieved for C-PSCs based on inorganic perovskites (inorganic C-PSCs) [22,23].

Among the different inorganic perovskites, inorganic CsPbI_3_ perovskite has the greatest application potential owing to its ideal bandgap and non-volatile nature [24], [25], [26], [27], [28], [29], [30]. However, the perovskite phases of CsPbI_3_ (*p*-CsPbI_3_: *α, β*, and *γ* phases) are not stable at room temperature, and tend to transition to a non-perovskite phase (*δ*-CsPbI_3_) [30], [31], [32], [33]. The phase transition considerably worsens in a humid atmosphere because water molecules catalyse the phase transition by introducing vacancies into the crystal lattice and lowering the free-energy barrier to nucleation [34]. As a result, most high-quality CsPbI_3_ perovskite films and corresponding PSCs are prepared in dry air or an inert atmosphere. Consequently, it is a research consensus that humidity or water is detrimental to CsPbI_3_ perovskite, and should be avoided during film and device processing.

However, our present findings have demonstrated that the aforementioned view on the effects of water on CsPbI_3_ perovskite does not always hold true. Herein, when the CsPbI_3_ precursor solutions were prepared in a humid atmosphere instead of a dry or inert atmosphere, higher film quality and device performance could be obtained. An in-depth analysis indicated that the incorporation of H_2_O in precursor solutions led to the generation of smaller colloidal particles, composed of low iodine coordination complexes, which retarded the crystallization of dimethylammonium lead iodide (DMAPbI_3_) intermediate. As a result, the conversion of DMAPbI_3_ to *p*-CsPbI_3_ was considerably accelerated, which enhanced the crystallinity of *p*-CsPbI_3_ and improved the orientation of the *p*-CsPbI_3_ crystals. Consequently, the C-PSCs based on the CsPbI_3_ achieved a PCE of 16.05%, which is a new record for inorganic C-PSCs, to the best of our knowledge.

## 2 Results and discussion

**Fig. 1:**
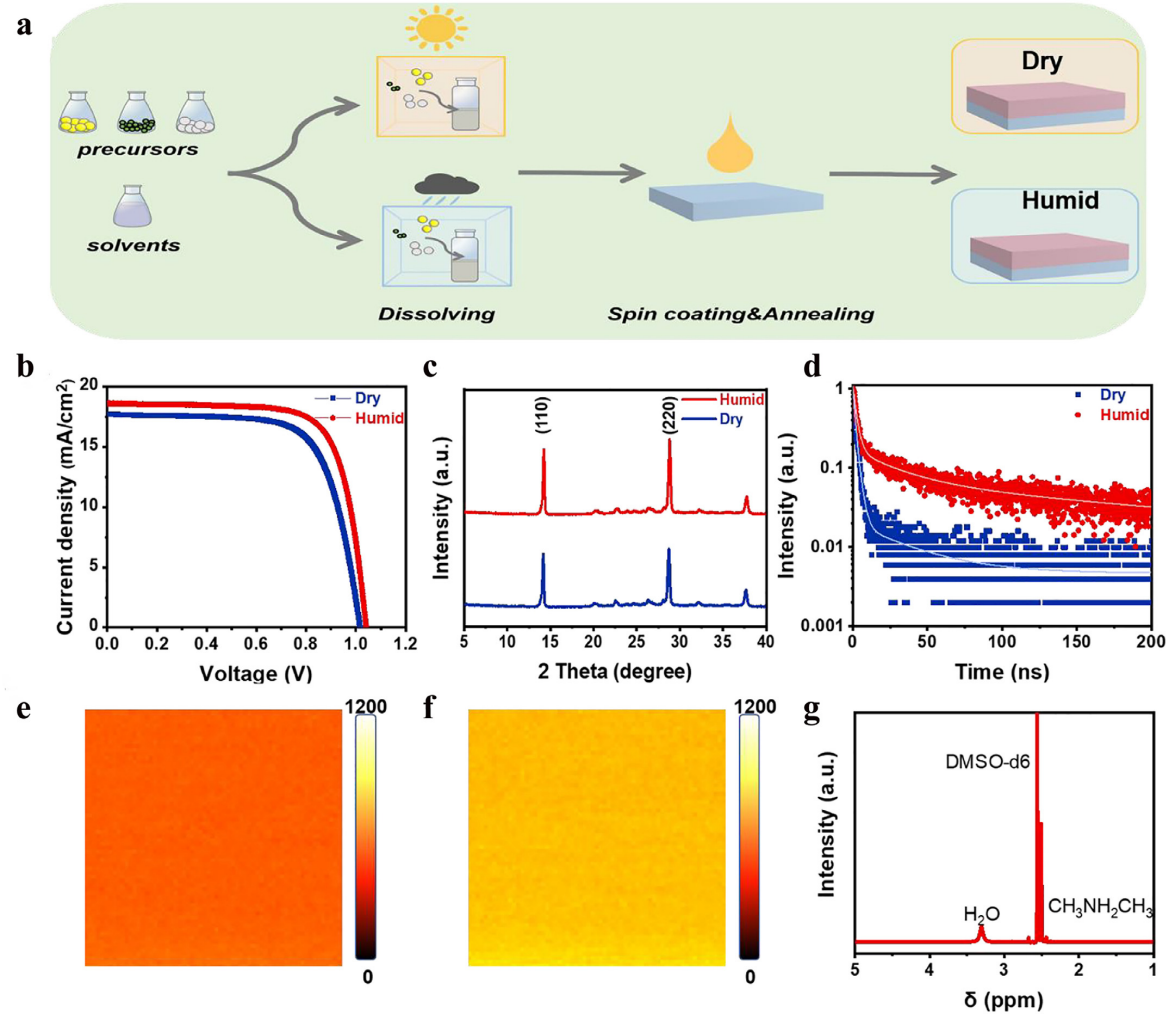
**Solution-processed CsPbI_3_ films under Dry or Humid environments.** (a) Schematic of the solution-processed CsPbI_3_ films under Dry or Humid environments. (b) *J-V* curves of the C-PSCs prepared from the Dry and Humid films; (c) XRD patterns and (d) TRPL spectra of Dry and Humid films; confocal PL intensity maps of (e) Dry and (f) Humid films; and (g) 1H NMR spectra of precursor salts (DMAI, CsI, and PbI_2_) after exposure to humid air. The powder for NMR measurement was dissolved in DMSO-d6.

The precursors used for the deposition of CsPbI_3_ films mainly included dimethylammonium iodine (DMAI), lead iodide (PbI_2_), and caesium iodide (CsI); dimethylformamide (DMF) was used as the solvent. All of the chemicals were stored in dry boxes. As illustrated in Fig. 1a, the precursor solutions were either prepared in a simple glove box (filled with dry air, RH < 1%) or in an ambient atmosphere (RH > 30%), denoted as ‘Dry’ or ‘Humid’, respectively. Both the deposition of CsPbI_3_ films by spin-coating and carbon electrodes by painting were conducted in a simple glove box (filled with dry air, RH < 1%). It was expected that the Humid C-PSCs would have lower performance than the Dry C-PSCs because a high humidity could induce a phase transition. However, it was found that the Humid C-PSCs exhibited obviously higher performance (PCE: 14.27%) than the Dry devices (PCE: 12.62%) (Fig. 1b).

To further understand the cause of the performance difference between the two devices, a series of measurements were conducted on the two CsPbI_3_ films. The X-ray diffraction (XRD) patterns in Fig. 1c reveal that the main diffraction peaks for the two films are similar. No diffraction peak associated with *δ*-CsPbI_3_ was detected. The intensive peaks at 14.3° and 28.8° correspond to the (110) and (220) planes of *p*-CsPbI_3_, respectively. An obvious enhancement in the peak intensity is seen for Humid films in comparison to Dry films, demonstrating the improved crystallinity of *p*-CsPbI_3_. The confocal photoluminescence (PL) intensity maps in Fig. 1e and f shows that the absolute PL intensity for the Humid film is much higher than that for the Dry film. Time-resolved PL (TRPL) spectra in Fig. 1d clearly show that the Humid film exhibits an obviously lower quenching rate (lifetime: 34.02 ns) than the Dry film (lifetime: 11.09 ns). Both PL intensity mapping and TRPL spectra reflect the reduced defect density in Humid films. Therefore, improvements in crystallization and reductions in defect density could account for the enhancement in device performance.

As stated above, all preparation processes for the Dry and Humid films were the same, except for the preparation atmosphere for the precursor solutions. Since the major difference between the two preparation atmospheres is the humidity, it is reasonable to infer that H_2_O could play an important role in the reaction because the precursor salts are hygroscopic and H_2_O is easily incorporated into them. To verify this, the precursor powders were deliberately exposed to humid air and the proton nuclear magnetic resonance (1H NMR) spectrum was recorded after dissolution in dimethyl sulfoxide-d6 (DMSO-d6) (Fig. 1g). The signal located at *δ* = 2.55 ppm corresponds to the protons in the -CH_3_ (doublet) group of DMAI, while the characteristic peak of H_2_O is also detected at *δ* = 3.33 ppm, indicating the absorption of H_2_O on the precursor salts during preparation in an ambient atmosphere.

**Fig. 2:**
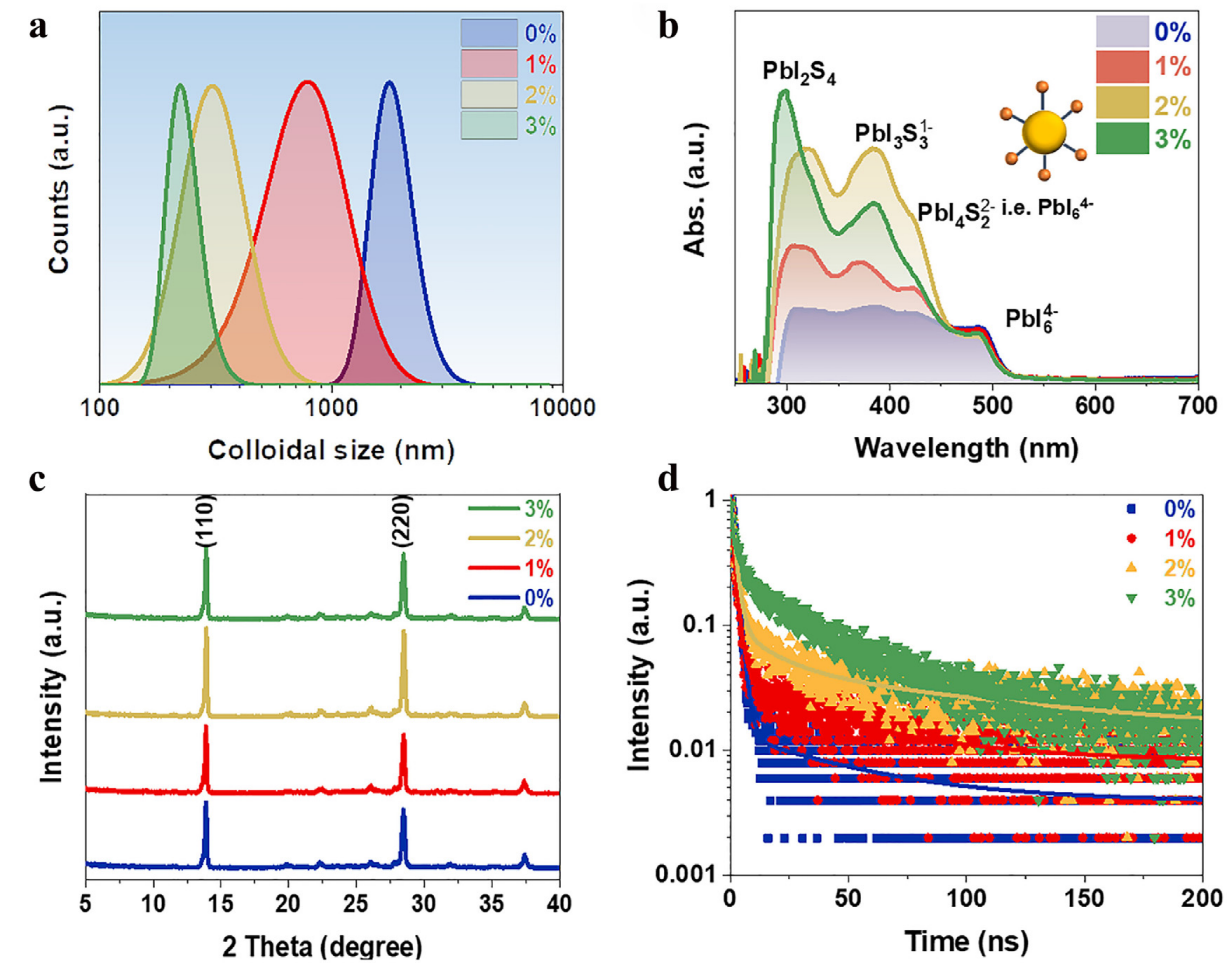
**Effects of H_2_O addition on precursor solutions and perovskite films.** (a) Colloid size distribution and (b) UV-Vis spectra of the precursor solutions with different H_2_O contents; (c) XRD patterns and (d) TRPL spectra of the perovskite films prepared from the precursor solutions with different H_2_O contents.

To verify the positive effects of H_2_O in precursor solution on the CsPbI_3_ perovskite and device performance, a small amount (0–3%) of H_2_O was directly added to the dry precursor solutions. As has been widely reported, most perovskite precursor solutions are colloidal solutions, and the colloidal properties – which would be largely determined by solution composition and the solvent itself – greatly influenced the nucleation and growth of perovskite films [35,36]. Therefore, the effects of H_2_O addition on the colloidal properties of the CsPbI_3_ precursor solutions were first studied using dynamic light scattering. As indicated in Fig. 2a, the average size of the colloidal particles in the pristine solution is about 1706 nm. After H_2_O addition, the average size gradually decreases with increases in the amount of H_2_O: 746 nm, 302 nm, and 217 nm for the 1%, 2%, and 3% H_2_O solutions, respectively.

For a more in-depth analysis, ultraviolet–visible (UV-Vis) spectra (Fig. 2b) were measured for the colloidal solutions. Multiple plateau absorption edges could be observed in the wavelength range from 300 to 550 nm, suggesting that a series of coordination compounds are formed in the solutions. As previously reported, these coordination compounds should be lead polyiodides, such as [PbI_2_S_4_], [PbI_3_S_3_]^1−^, [PbI_4_S_2_]^2−^, and [PbI_6_]^4-^ (‘S’ represents solvent molecule) [37]. In the precursor solution, the solvent competes with iodide ions to coordinate with lead ions, which dictates the type of complexes present in the precursor solutions. For the bare precursor solution, four absorption peaks with comparable intensities could be observed, indicating the coexistence of the four coordination compounds. The absorption peaks of [PbI_2_S_4_], [PbI_3_S_3_]^1−^, and [PbI_4_S_2_]^2−^ are gradually enhanced as the H_2_O content increases to 2%, accompanied by the weakened peak of [PbI_6_]^4–^. With the H_2_O content increasing to 3%, the absorption peak of [PbI_2_S_4_] becomes dominant, while all other peaks are weakened. Therefore, it can be concluded that low iodine coordination complexes form preferentially after H_2_O addition, which could be due to the higher coordinating ability of H_2_O than iodide ions. It is widely known that the coordination compounds in precursor solutions have a significant influence on the crystallization of perovskite films, and it is desirable to obtain full iodine coordination complex colloids with a perovskite structure, as it provides a similar coordinating environment as the crystal structure, which is perovskite. It is expected that the addition of H_2_O in the CsPbI_3_ precursor solution (DMAI, PbI_2_, and CsI) would induce an increase in the low iodine coordination complexes, which would strongly lower perovskite (DMAPbI_3_) crystallization. Such relations between solvent coordination and perovskite crystallization have been widely observed in the formation of MAPbX_3_ [38], [39], [40], [41], [42].

The abovementioned precursor solutions were used to prepare CsPbI_3_ films by spin-coating, followed by annealing at 220 °C. The XRD patterns in Fig. 2c exhibit obvious diffraction peaks at 14.3°, 20.27°, and 28.8° for all films, corresponding to the (110), (020), and (220) planes of *p*-CsPbI_3_, respectively. Therefore, the addition of H_2_O lower than 3% has a negligible influence on the phase structure. Interestingly, the addition of H_2_O into the precursor solution enhances the peak intensity of *p*-CsPbI_3_ well, especially at 2%, proving that a moderate amount of H_2_O could help to enhance the crystallinity of *p*-CsPbI_3_. The TRPL spectra in Fig. 2d show that the addition of H_2_O slows down the quenching rate of PL, and the average lifetimes for the 0%, 1%, 2%, and 3% films are calculated to be 1.93 ns, 11.64 ns, 23.54 ns, and 26.14 ns, respectively. This indicates that the reduced defect density suppresses charge recombination. As a side note, the enhancement in PL lifetime is more obvious than that in XRD peak intensity. This may be because XRD measures the crystallinity of the entire perovskite film, while the TRPL lifetime is predominantly affected by the surface traps of perovskite grains. The addition of H_2_O could have reduced trap density more efficiently at the surface than in the inner regions of the perovskite grains. Overall, the addition of H_2_O in the precursor solution greatly affects the colloidal properties, enhances crystallization, and reduces defect density.

**Fig. 3:**
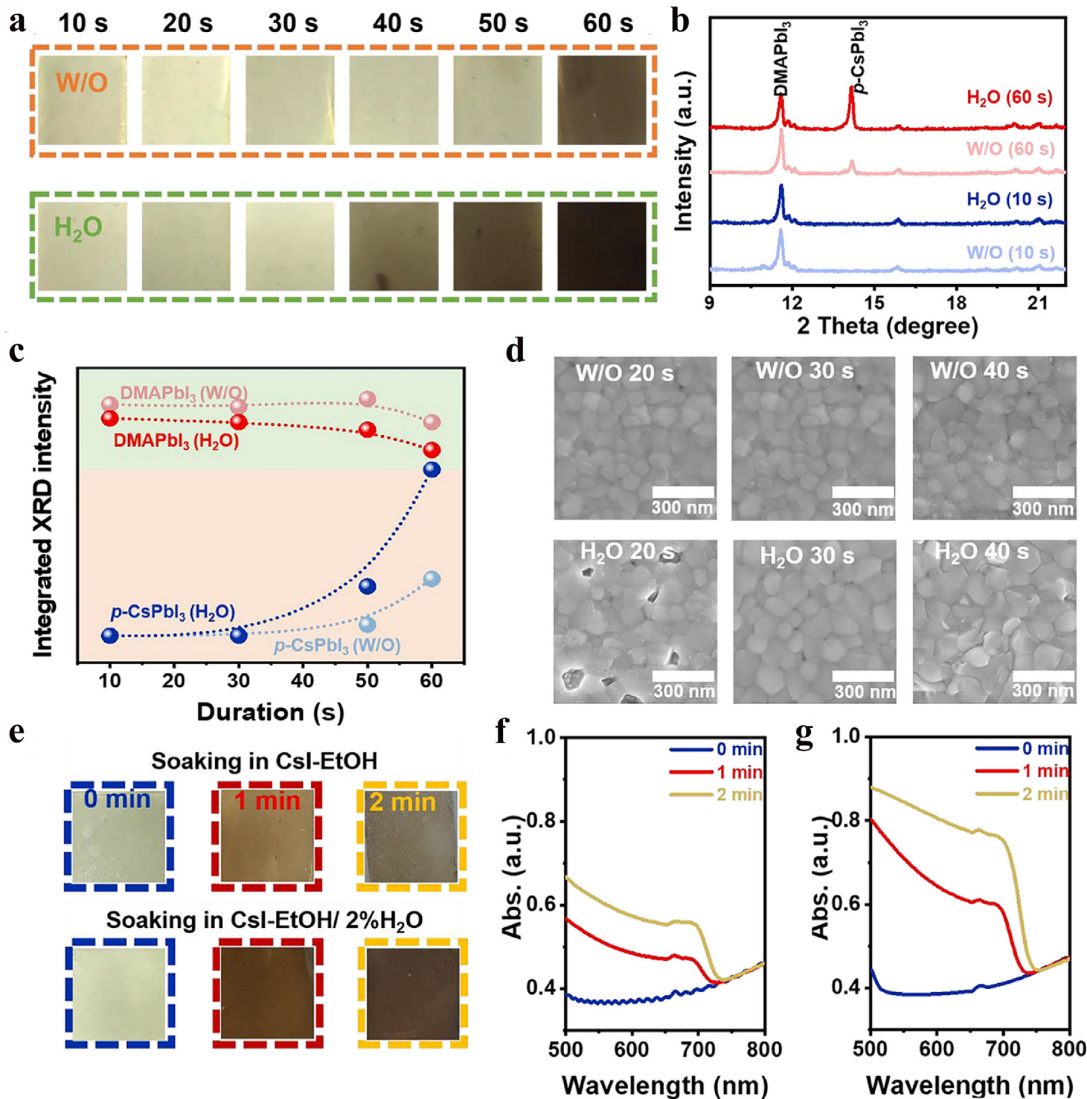
**Mechanism of the H_2_O-promoted growth of *p*-CsPbI_3_.** (a) Photographs of the W/O and H_2_O films after annealing for periods (10–60 s). (b) XRD patterns of the W/O and H_2_O films after annealing for 10 and 60 s. (c) Change in the integrated intensity of the DMAPbI_3_ (11.6°) and *p*-CsPbI_3_ (14.3°) diffraction peaks with annealing time for the W/O and H_2_O films. (d) SEM images of the W/O and H_2_O films after annealing for different periods. (e) Photographs and (f, g) UV-Vis spectra of the DMAPbI_3_ films after soaking in CsI-EtOH and CsI-EtOH/2% H_2_O solutions for periods.

To determine the cause for the better crystal quality, the effects of H_2_O addition on the crystallization kinetics of the CsPbI_3_ films were further investigated by comparing 0% (W/O) and 2% H_2_O (H_2_O) samples. Photographs (Fig. S1) and XRD patterns (Fig. S2) of the films obtained at different annealing (220 °C) stages were collected. Overall, after the addition of H_2_O, the conversion of the DMAPbI_3_ intermediate to *p*-CsPbI_3_ is accelerated, while the transition of *p*-CsPbI_3_ to *δ*-CsPbI_3_ is retarded. For a deeper understanding of the conversion process, the initial 60 s of the annealing process is studied in detail. As shown in Fig. 3a, the film changes from yellowish to light brown at around 60 s for the W/O film, while this occurs at about 40 s for the H_2_O film – a darkened film is obtained at 60 s – which confirms the accelerated conversion of DMAPbI_3_ to *p*-CsPbI_3_ after the addition of H_2_O.

XRD patterns (Fig. S3 and Fig. 3b) show that DMAPbI_3_ is formed first, and is then converted into *p*-CsPbI_3_ in both cases. For the H_2_O film, the conversion is obviously accelerated, which is consistent with the observations from the photographs. To visually describe the change of the DMAPbI_3_ and *p*-CsPbI_3_, the integrated intensities of the diffraction peaks of DMAPbI_3_ (11.6°) and *p*-CsPbI_3_ (14.3°) were extracted from the XRD patterns and plotted in Fig. 3c. It is apparent that the peak intensity of DMAPbI_3_ is weakened after the addition of H_2_O during the initial 60 s, which indicates the lower crystallinity under the effect of H_2_O. This phenomenon may be related to the formation of smaller colloidal particles with lower iodine coordination in the precursor solution, which hinders the rapid crystallization of DMAPbI_3_. Moreover, the DMAPbI_3_ peak intensity exhibits a more obvious downward trend for the H_2_O film than that for the W/O film, accompanied by a more rapid increase in the peak intensity of *p*-CsPbI_3_. It can be concluded that the addition of H_2_O lowers the crystallinity of DMAPbI_3_ and considerably accelerates the conversion of DMAPbI_3_ to *p*-CsPbI_3_. The top-view scanning electron microscope (SEM) images in Fig. 3d and Fig. S4 indicate that there are many pinholes in the H_2_O film at the initial annealing stage, when the film is mainly composed of DMAPbI_3_, while the W/O film is almost pinhole-free, further confirming the lowered crystallinity of DMAPbI_3_ under the effect of H_2_O. In addition, at 40 s of annealing, some newly formed grains are observed for the H_2_O film, which is associated with the formation of *p*-CsPbI_3_ crystals.

As stated above, H_2_O could weaken the crystallization of DMAPbI_3_, accelerating the conversion of DMAPbI_3_ to *p*-CsPbI_3_. Owing to this, it is important to ascertain whether H_2_O only accelerates the disassociation of DMAPbI_3_ crystals or even removes DMAI from the film. Therefore, X-ray photoelectron spectroscopy (XPS) was used to evaluate the composition change of the W/O and H_2_O films during annealing (Fig. S5). It can be seen that the N 1s peak at 402.2 eV gradually weakens with annealing time due to the volatilization of DMAI. However, no difference in the weakening trend is observed between the two films, and the contents of DMAI residuals in both films are similar throughout the process. Therefore, it can be concluded that H_2_O does not help remove DMAI from the film, but only accelerates the disassociation of DMAPbI_3_ crystals to promote the conversion.

To further confirm the above-mentioned analysis, we simulated the processes of DMAPbI_3_ losing DMAI and reacting with Cs-I to form *p*-CsPbI_3_. A pure DMAPbI_3_ film was used as the precursor film, while a CsI-EtOH solution was used as the reaction solution. After soaking the DMAPbI_3_ film in the CsI-EtOH solution, the yellowish film gradually becomes brown within 2 min, implying the conversion of DMAPbI_3_ to *p*-CsPbI_3_ (Fig. 3e and Fig. S6). The UV-Vis spectra in Fig. 3f confirm the generation of *p*-CsPbI_3_, as the absorption onset at around 730 nm gradually enhances. The XRD patterns in Fig. S7 show the gradual weakening of the diffraction peaks of DMAPbI_3_ after the film was soaked in CsI-EtOH solution. After 2% H_2_O was added to the CsI-EtOH solution, the yellowish film became brown more rapidly, demonstrating that the conversion was accelerated, as confirmed by the UV-Vis spectra in Fig. 3g. More importantly, the diffraction peaks of DMAPbI_3_ were weakened more pronounced after the addition of 2% H_2_O, which supports the fact that H_2_O promotes the dissociation of DMAI from DMAPbI_3_ to induce the faster generation of *p*-CsPbI_3_.

**Fig. 4:**
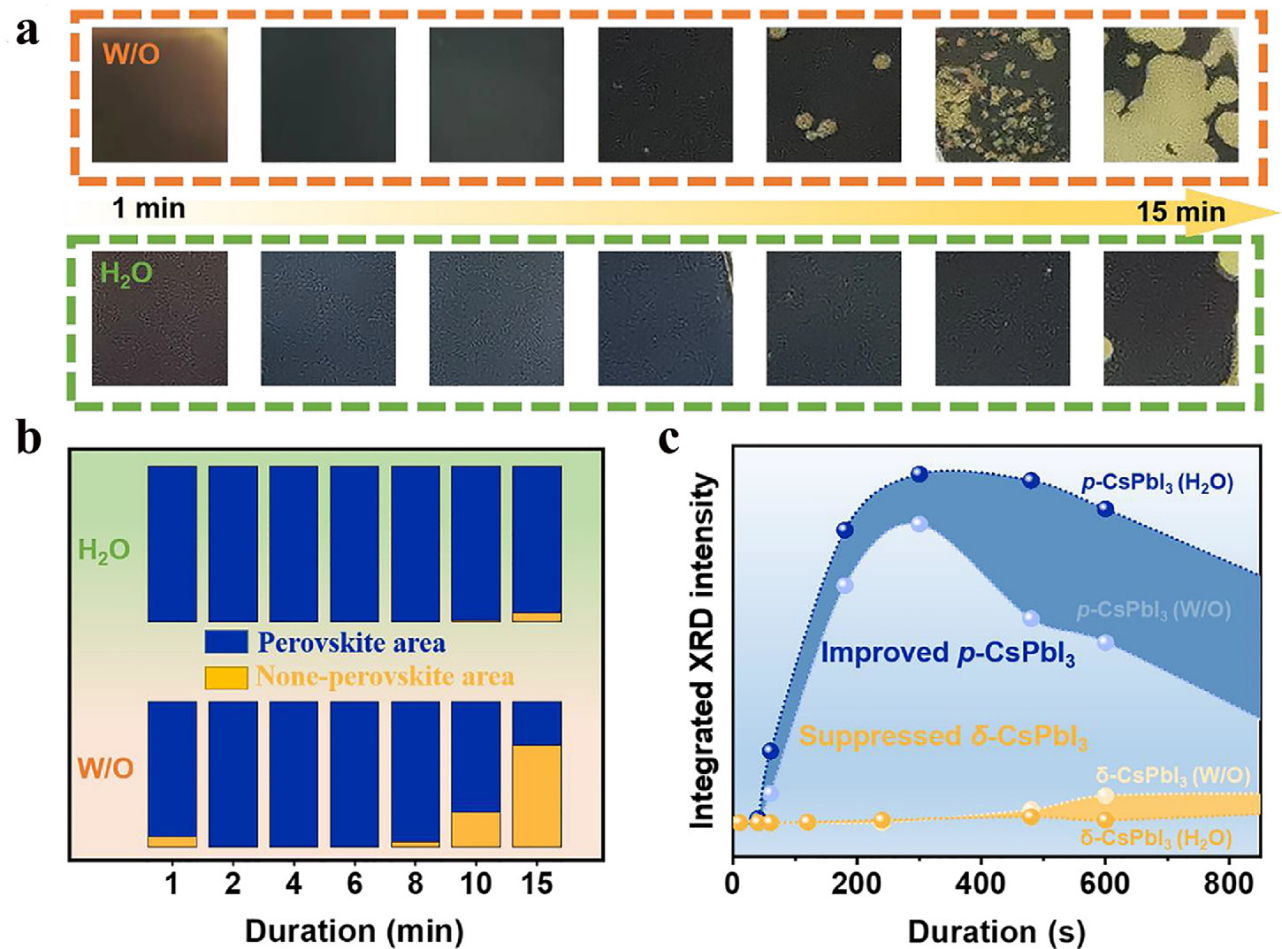
**Phase evolution of the W/O and H_2_O films with annealing time (1 to 15 min).** (a) Photographs of the W/O and H_2_O films at different annealing times, (b) comparison of the perovskite (blue) and non-perovskite (yellow) areas obtained from the corresponding binary images of the photographs in (a), and (c) the change of the integrated intensity of the diffraction peaks of *δ*-CsPbI_3_ (9.0^o^) and *p*-CsPbI_3_ (14.3^o^) for the W/O and H_2_O films with annealing time.

At the later annealing stages, both films become blacker as more and more DMAPbI_3_ is converted to *p*-CsPbI_3_, which is supported by the XRD patterns in Fig. S2. Therefore, too long an annealing time would induce the transition of *p*-CsPbI_3_ to *δ*-CsPbI_3_. However, obvious differences can also be observed between the W/O and H_2_O films at this stage. As shown in Fig. 4a, the W/O films become black slower but turn yellow more quickly than the H_2_O films. To roughly quantify the area of the dark and yellow (or perovskite and non-perovskite) regions, the photographs in Fig. 4a were transformed into a black-and-white binary image (Fig. S8), and the perovskite and non-perovskite region were calculated (Fig. 4b). After 1 min of annealing, some area still does not become brown in the W/O films, while the entire H_2_O film appears brown, indicating that the conversion is slower for the former. As the annealing duration prolongs, the W/O films appear yellow much earlier (around 8 min) than the H_2_O films (around 15 min), implying the easier conversion of *p*-CsPbI_3_ to *δ*-CsPbI_3_ for the former. After annealing for 15 min, the yellow area of the W/O films gradually enlarged to 70.2%, while the yellow area for the H_2_O films is only 5.6%. Therefore, the *p*-CsPbI_3_ film obtained under the effects of H_2_O is more stable than the pristine one.

To better interpret this analysis, the integrated intensities of the diffraction peaks of *δ*-CsPbI_3_ (9^o^) and *p*-CsPbI_3_ (14.3^o^) for the W/O and H_2_O films at different annealing times are obtained from the XRD patterns. As plotted in Fig. 4c, within the first 5 min, the peak intensity of *p*-CsPbI_3_ gradually increases for the W/O films, and no *δ*-CsPbI_3_ peak is detected. With the duration further increasing, the peak intensity of *p*-CsPbI_3_ begins to decrease rapidly, accompanied by the continuous enhancement in the peak intensity of *δ*-CsPbI_3_. In comparison, the H_2_O films exhibit a more rapid increase in the peak intensity of *p*-CsPbI_3_ than the W/O films within the first 5 min, and the intensity remains almost unchanged as the duration increases to 10 min. The observable peak intensity of *δ*-CsPbI_3_ could only appear at 15 min for the H_2_O films, but it appears at 8 min for the W/O films, demonstrating the enhancement in the phase stability of the former very well. The higher crystallite and more compact morphology could account for the improved phase stability. Therefore, it is clear that the addition of H_2_O not only improves the crystallinity of *p*-CsPbI_3_ but also enhances its phase stability. Most importantly, the time window for the processing of high-quality *p*-CsPbI_3_ films is widened, which is highly desired for industrial production.

**Fig. 5:**
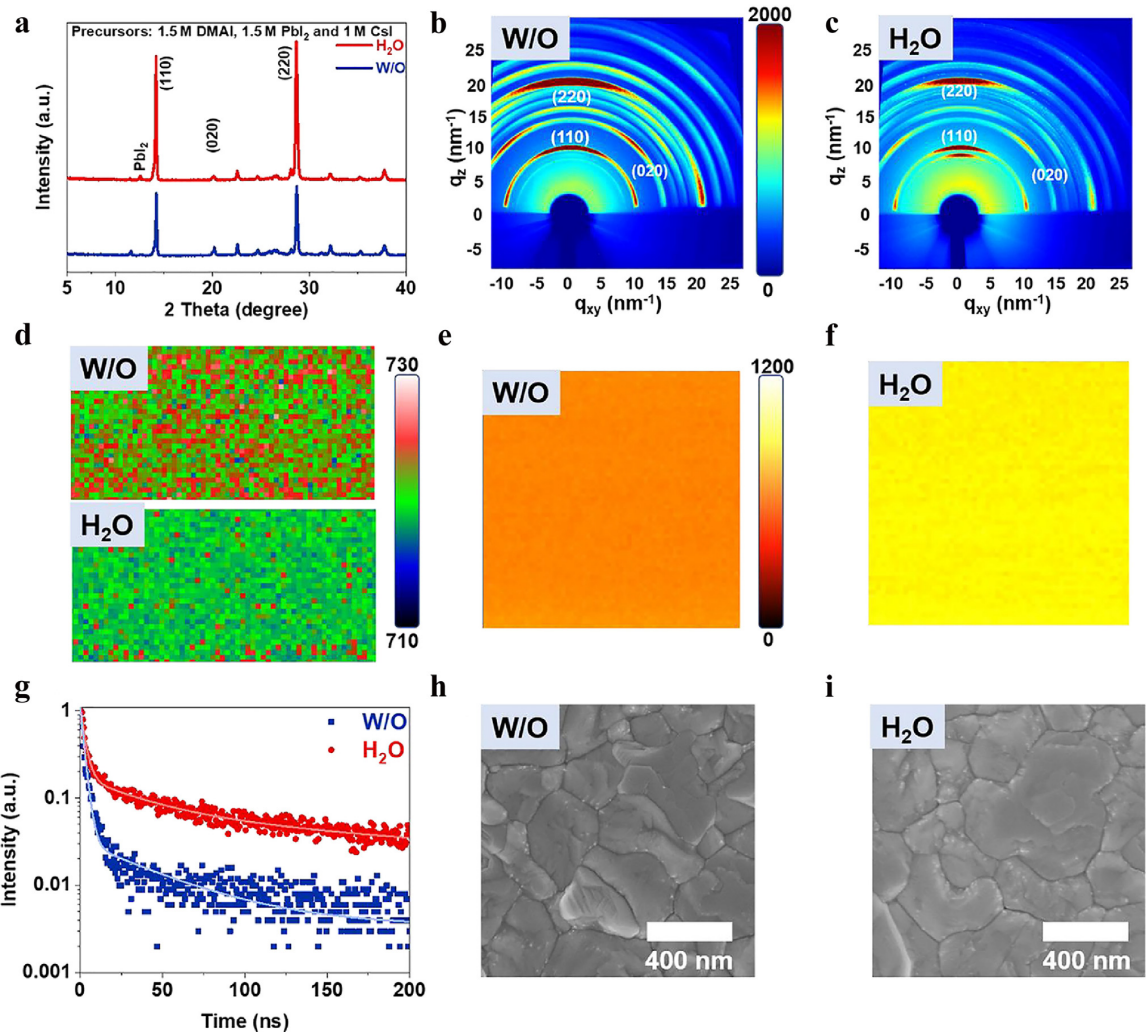
**Comparison between the W/O and H_2_O films prepared from the precursor solutions containing 1.5 M DMAI, 1.5 M PbI_2_, and 1 M CsI.** (a) XRD patterns, (b, c) GIWAX images, (d) confocal PL peak position maps, (e, f) confocal PL peak intensity maps, (g) TRPL spectra, and (h, i) SEM images.

These results demonstrate that the addition of H_2_O in the basic CsPbI_3_ precursor solution (1 M DMAI, 1 M PbI_2_ and 1 M CsI) improves the quality of *p*-CsPbI_3_ films. Our previous work has demonstrated that extra PbI_2_ in CsPbI_3_ film could passivate defects and improve the film quality and device performance; further, the optimized solution contains 1.5 M DMAI, 1.5 M PbI_2_, and 1 M CsI. To further improve film quality, H_2_O was also added to the optimized precursor solution. As expected, the addition of H_2_O also greatly enhances the crystallinity of CsPbI_3_, which is confirmed by the increased peak intensity in the XRD pattern (Fig. 5a). In addition, the diffraction peak of PbI_2_ at 12.6° is obviously enhanced, while the diffraction peak of DMAPbI_3_ at 11.6° is largely weakened, which supports the fact that DMAPbI_3_ is more easily dissociated under the effect of H_2_O, generating PbI_2_ residuals. The increase in PbI_2_ residuals more effectively passivates defects and enhances film quality. Furthermore, it can be found that the peak intensity ratio of (110)/(020) obviously increases, from 9.13 to 25.23, after H_2_O addition, indicating that the presence of H_2_O has induced the preferable growth of the (110) crystallographic planes for *p*-CsPbI_3_. Grazing-incidence wide-angle X-ray scattering (GIWAXS) was further employed to assess the crystal orientation. As shown in Fig. 5b and c, the H_2_O sample exhibits much sharper and more discrete diffraction rings in the (110) and (220) planes, which further confirms a preferred (110) orientation for the *p*-CsPbI_3_ grains.

The confocal PL peak position maps in Fig. 5d show that the PL peak positions are similar for both films – in the range of 710 to 730 nm – corresponding to the fluorescence of *p*-CsPbI_3_. However, the H_2_O films exhibit a much more unified distribution, implying a more uniform perovskite phase. Confocal PL intensity maps (Fig. 5e,f) visualize the significant enhancement in the absolute PL intensity of the H_2_O film in comparison to the W/O film. The TRPL spectra in Fig. 5g clearly show the slower quenching rate for the H_2_O film than that for the W/O film. After fitting, the average lifetimes for the W/O film and H_2_O films are 6.82 and 37.39 ns, respectively. Therefore, the addition of H_2_O in the optimized solution also reduces the defect density of the *p*-CsPbI_3_ film well. SEM images in Fig. 5h,i indicate that the H_2_O film is slightly more compact and more uniform than the W/O film. In summary, H_2_O addition has greatly enhanced the crystallinity of the CsPbI_3_ film with a low defect density and improved the morphology.

**Fig. 6:**
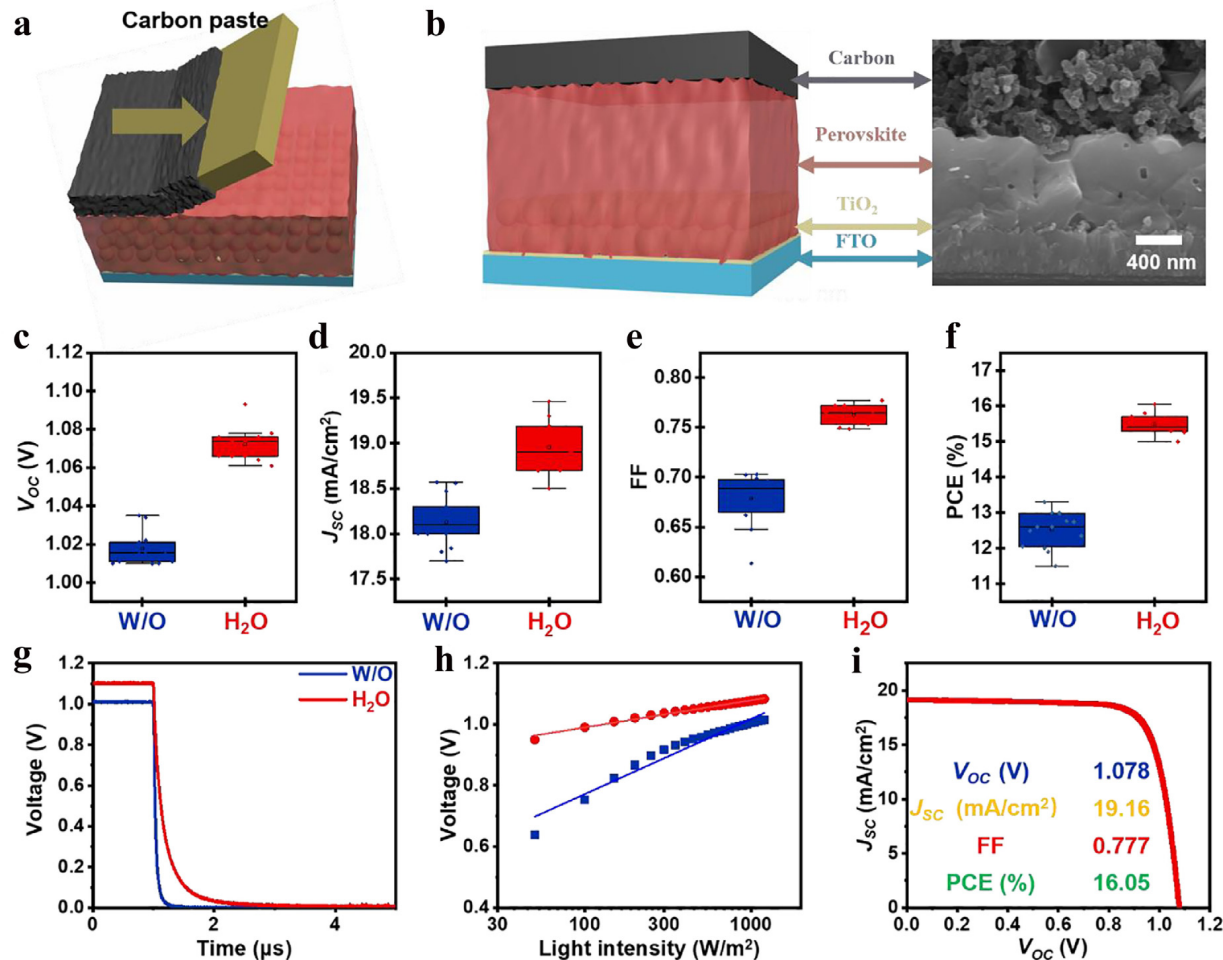
**Device structure and performance comparison between the W/O and H_2_O C-PSCs.** (a) Schematic illustrating the deposition of the carbon electrode on the perovskite film using the doctor-blade method and (b) scheme and cross-sectional SEM image of C-PSCs. Statistical data obtained from the W/O and H_2_O C-PSCs: (c) *V_OC_*, (d) *J_SC_*, (e) FF, and (f) PCE. (g) TPV and (h) dependence of *V*_*OC*_ on *I* for the W/O and H_2_O C-PSCs. (i) J-V curve of the best H_2_O C-PSCs.

To fabricate C-PSCs, commercial carbon paste was directly painted on the *p*-CsPbI_3_ films (Fig. 6a). A cross-sectional SEM image of the C-PSCs (Fig. 6b) shows the close contact at the CsPbI_3_/carbon interface. The statistical photovoltaic metrics in Fig. 6c-f show that the H_2_O devices exhibit greatly enhanced performance in comparison to the W/O devices, with the PCE increasing from 12.5 ± 1% to 15.5 ± 0.5%. This enhanced PCE is attributed to the overall improvement in photovoltaic parameters. The average *V_OC_, J_SC_*, and FF for the W/O devices are only 1.017 ± 0.017 V, 18.2 ± 0.4 mA/cm^2^, and 0.658 ± 0.045, while the values are improved to 1.077 ± 0.016 V, 19 ± 0.5 mA/cm^2^, and 0.763 ± 0.015, respectively, for the H_2_O devices.

The transient photovoltage decay (TPV) results (Fig. 6g) show that the H_2_O device has a longer charge-carrier lifetime than the W/O device, which indicates that the carrier recombination is greatly suppressed in the H_2_O device [43, 44]. The light intensity (*I*) dependences of *J_SC_* and *V_OC_* are shown in Fig. S9 and Fig. 6h, respectively. The recombination mechanism can be understood according to Eqs. 1,2 [45], where *k* is the Boltzmann constant, *T* is the absolute temperature, *q* is the elementary charge, and *α* and *ε* are ideal factors related to recombination.

(1) $$\begin{array}{c} J_{\mathrm{SC}}\;\propto \;I^{\alpha }\;\;\left(\alpha \;\leq \;1\right)\; \end{array}$$

(2) $$\begin{array}{c} V_{\mathrm{OC}}\;=\;\varepsilon \mathrm{kTln}\left(I\right)/q\;+\;\mathrm{cons}\mathrm{tant} \end{array}$$

The *V_OC_*-*I* curves show that the *V_OC_* increases with *I*; the *ε* for the H_2_O device is 2.9, which is lower than that for the W/O device (5.8), indicating reduced trap-assisted recombination. A linear dependence of *J_SC_* on *I* is found for both devices, and the *α* values for both devices are over 0.95, demonstrating an effective charge separation in both C-PSCs [46]. The transient photocurrent decay (TPC) in Fig. S10a shows that the decay time for the H_2_O device slightly decreases in comparison to the W/O device, which may benefit from the better orientation of the H_2_O films. As a result, the best CsPbI_3_ C-PSCs display a *V_OC_* of 1.078 V, *J_SC_* of 19.16 mA/cm^2^, and FF of 0.777, which yields a PCE of 16.05% (Fig. 6i), a new record PCE for inorganic C-PSCs, to the best of our knowledge. The *J-V* curves obtained from forward and reverse scan directions (Fig. S10b) and the stay-state maximum power output (Fig. S10c) reveal that our device has a small amount of hysteresis and a stable power output. After storing the non-encapsulated device in a dry air atmosphere for 100 days, almost no PCE degradation was observed (Fig. S10d), demonstrating its high stability.

## 3 Conclusion

We have demonstrated that the incorporation of H_2_O in precursor solutions (from humid air or by intentional addition) was beneficial to the growth of high-quality CsPbI_3_ perovskite. The presence of H_2_O in the precursor solution considerably increased the amount of low iodine coordination complexes, which suppressed the crystallization of the DMAPbI_3_ intermediate. The DMAPbI_3_ intermediate, with low crystallinity, facilitated the subsequent conversion to CsPbI_3_ perovskite with a higher orientation, improved the crystallinity, and lowered the defect density, which raised the PCE of CsPbI_3_ C-PSCs to 16.05%, a new record for inorganic C-PSCs, to the best of our knowledge.

## Declaration of competing interest

The authors declare that they have no conflicts of interest in this work.

## Appendix Supplementary materials

Image, application 1
