# Supplementary material for: Moisture is not always bad: H2O accelerates the conversion of DMAPbI3 intermediate to CsPbI3 for boosting the efficiency of carbon-based perovskite solar cells to over 16%
Source: Fundam Res. 2022 Jul 24;4(5):1110–7. doi: 10.1016/j.fmre.2022.07.005 (PMC11489495; doi:10.1016/j.fmre.2022.07.005)
Supplement: Supplementary file 1 [file mmc1.docx]

**Moisture is not alway bad: H_2_O accelerates the conversion of DMAPbI_3_ intermediate to CsPbI_3_ for boosting the efficiency of carbon-based perovskite solar cells to over 16%**

Hailiang Wang^a^, Huicong Liu^a^, Zijing Dong^a^, Xueyuan Wei^b^, Weiping Li^a^, Liqun Zhu^a^, Cheng Zhu^b^, Yang Bai^b^, Haining Chen^a^*

^a^ School of Materials Science and Engineering, Beihang University, No. 37 Xueyuan Road, Haidian District, Beijing 100191, People’s Republic of China.

^b^ Beijing Key Laboratory of Construction Tailorable Advanced Functional Materials and Green Applications, MIIT Key Laboratory for Low-dimensional Quantum Structure and Devices, Experimental Center of Advanced Materials, School of Materials Science & Engineering, Beijing Institute of Technology, Beijing 100081, People’s Republic of China.

Supporting information

**Materials and Methods**

Materials:

CsI (99.9%), PbI_2_ (99.99%) and DMAI (99.5%) was purchased from Xi'an Polymer Light Technology Corp. DMF (99.8%, extra dry) was purchased from Acros Organics. Titanium diisopropoxide (75 wt. % in isopropanol) were purchased from TCI(Shanghai)Development Co., Ltd.. Commercial TiO_2_ paste (30 NR-D) was purchased from Dyesol company. 1-butanol (99%) and ethanol (99.5%) were purchased from Shanghai Aladdin Biochemical Technology Co. Ltd.. All chemicals were used as-received without further purification.

**Device fabrication.**

**Deposition of TiO_2_ scaffolds**

FTO glass was cleaned with successive sonication in deionized water, ethyl alcohol and iospropanol. TiO_2_ blocking layer was then spin coated onto FTO glass at 2000 rpm for 20 s, using a titanium diisopropoxide bis(acetylacetonate) solution in 1-butanol (0.15 M), and then heated at 120 °C for 5 min. TiO_2_ mesoporous scaffolds were deposited by spin coating at 5000 rpm for 30 s using a commercial TiO_2_ paste dispersed in ethanol, followed by sintering at 100 °C for 5 min and then at 550 °C for 30 min.

**Deposition of CsPbI_3_ perovskite film**

The CsPbI_3_ perovskite films were prepared by solution-based processes using DMAI, PbI_2_, CsI as precursor salts and DMF as solvent. The precursor solution was prepared by dissolving 1 M DMAI, 1 M PbI_2_ and 1 M CsI in DMF. All these chemicals are stored in dry boxes. The precursor solutions may be prepared in a simple glove box (filled with dry air, RH < 1%) or in ambient atmosphere (RH > 30%), denoted as ‘Dry’ or ‘Humid’. After that, both the Dry or Humid films were deposited by spin-coating at 2000 rpm for 20 s and annealing at 220 °C for around 5 min, which were conducted in a simple glove box (filled with dry air, RH < 1%).

The CsPbI_3_ perovskite solutions with different H_2_O concentrations were prepared by dissolving 1 M DMAI, 1 M PbI_2_ and 1 M CsI in DMF in the simple glove box. H_2_O (0%, 1%, 2% and 3%) was directly added into the solutions. Then, CsPbI_3_ films were deposited by spin coating the above solution onto substrate, followed by annealing at 220 °C.

For converting DMAPbI_3_ film to CsPbI_3_ films in CsI solutions, the DMAPbI_3_ films were deposited by spin coating the DMAPbI_3_ precursor solution (1 M PbI_2_ and 1 M DMAI in DMF) on substrate at 2000 rpm for 20 s, followed by annealing at 100 ℃ for 5 min. After cooling down to room temperature, the DMAPbI_3_ films were immersed in a CsI-EtOH or CsI-EtOH/2% H_2_O solutions for different durations. This conversation was conducted in dry air atmosphere.

To obtain the CsPbI_3_ film with PbI_2_ passivator, an optimized solution contained 1.5 M DMAI, 1.5 M PbI_2_ and 1 M CsI was prepared in the glove box to obtain W/O films. To further improve film quality, H_2_O was also added into the optimized precursor solution to obtain H_2_O films. Both the W/O or H_2_O films were prepared by spin coating the precursor solution on substrate followed by annealed at 220 °C.

**Deposition of carbon electrodes**

CsPbI_3_ C-PSCs were fabricated by directly painting commercial carbon paste on the perovskite films, followed by annealing at 100 ℃. The preparation of precursor solution was under humid or dry air atmosphere as specifically mentioned, other preparation processes of C-PSCs were carried out in dry air.

**Characterizations:**

X-ray diffraction (XRD) patterns were obtained by a Rigaku D/MAX-2500 X-ray diffractometer with an X-ray tube Cu Ka radiation. (λ =1.5406 Å). Two-dimensional synchrotron radiation GIWAXS was performed at Shanghai synchrotron Radiation Facility to analyze the crystallinity and orientation of the perovskite films. The optical absorption spectra of perovskite films were detected using a Shimadzu UV-3600 UV-vis spectrometer. Dynamic light scattering analysis was performed on a NanoBrook Omni instrument (Bruker) to study the precursor chemistry. PL mapping measurements were conducted by a Laser Scanning Confocal Microscope (Enlitech, SPCM-1000) in air. The excitation wavelength was 450 nm, and we collected the emission signal in the wavelength range from 375 nm to 1025 nm. Chemical states of film surface were evaluated by an X-ray photoemission spectroscopy (XPS, ESCALab250Xi). Scanning electron microscopy (SEM) images were obtained on a SUPRA55 SEM at an accelerating voltage of 5 kV. The proton nuclear magnetic resonance (1H NMR) spectrum was recorded using Bruker AVANCE Ⅲ 600M instrument. The precursor powders were deliberately exposed to humid air and then dissolved in dimethyl sulfoxide-d6 (DMSO-d6) before test. Time-resolved PL (TRPL) spectra taken on an ultrafast lifetime Spectrofluorometer (Delta flex) and a 475 nm ultrafast laser was used as the excitation light source.

The photovoltaic performance was tested under a solar light simulator (Newport Oriel Sol 3A, model number 94063A, AM 1.5 global filter) in ambient air. The light intensity was calibrated to 1 Sun (100 mW/cm^2^) using an Oriel reference solar cell (monocrystalline silicon) and meter. Current density-voltage (J-V) curves, dependence of J_sc_ and V_oc_ on light intensity, TPC and TPV curves were measured on ZENNIUM pro electrochemical workstation (ZAHNER-Elektrik GmbH & Co., KG, Germany). The active area of cells was masked at around 6.25 mm^2^. The scanning speed of *J-V* curves was 100 mV/s for both forward and reverse scans.

The device stability tests were performed by storing non-encapsulated devices in air atmosphere (RH ~ 10-20%) at room temperature (10-20 °C). *J-V* curves were measured and recorded periodically in ambient air atmosphere (RH~30-80%) to track the device performance.


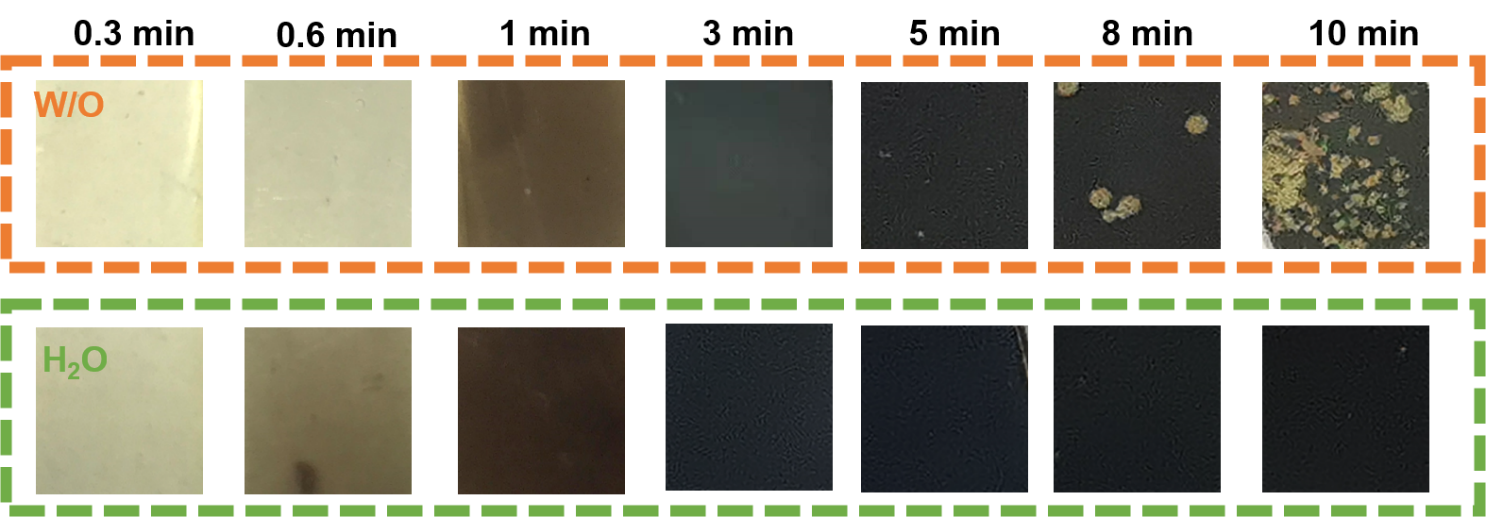


**Fig. S1.** Photographs of the W/O and H_2_O CsPbI_3_ films after annealing for different duration.


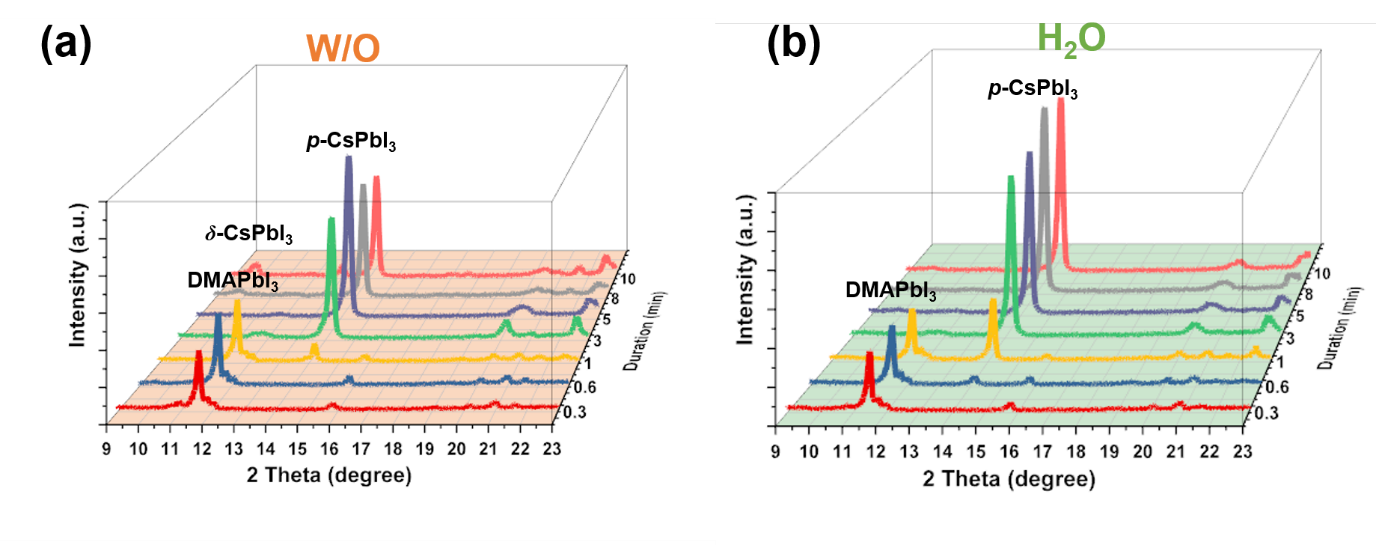


**Fig. S2.** XRD patterns of the (a) W/O and (b) H_2_O CsPbI_3_ films after annealing for different duration.


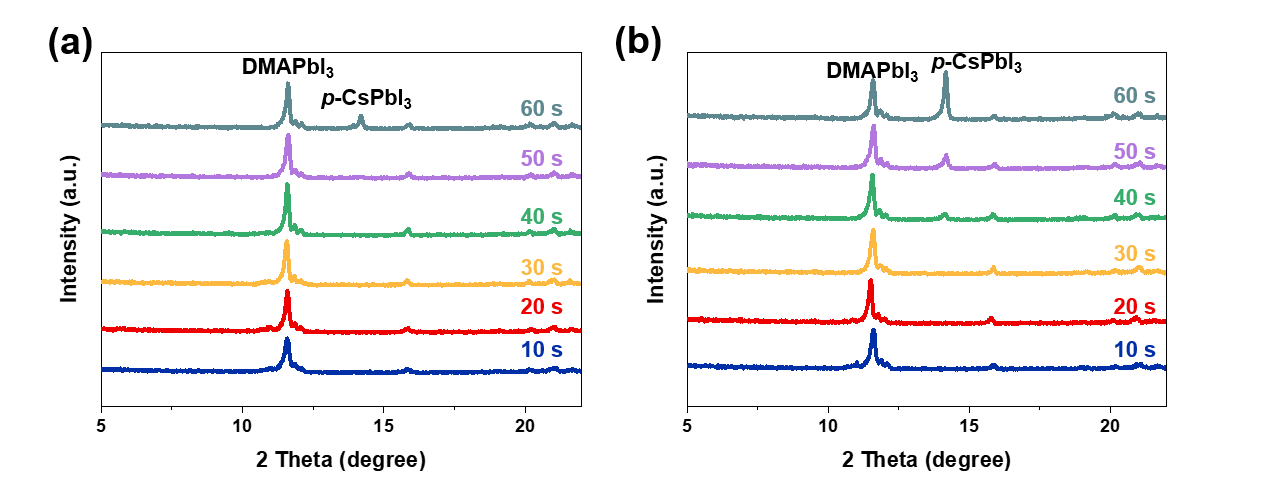


**Fig. S3.** XRD patterns of the (a) W/O and (b) H_2_O CsPbI_3_ films after annealing for different duration (10-60 s).


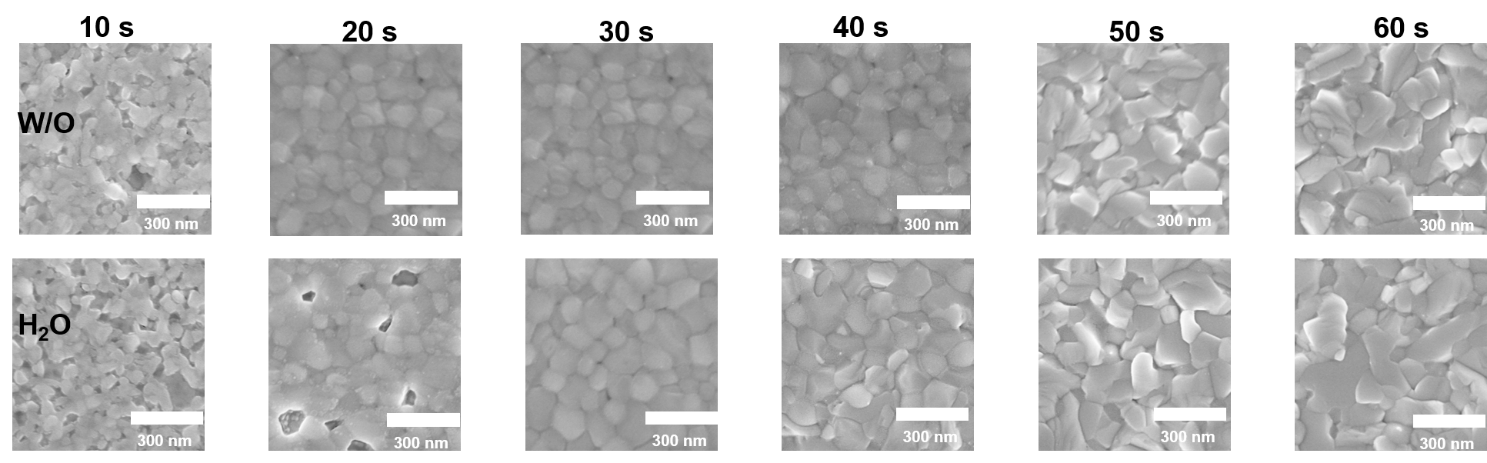


**Fig. S4.** SEM images of the W/O and H_2_O CsPbI_3_ films after annealing for different duration (10-60 s).


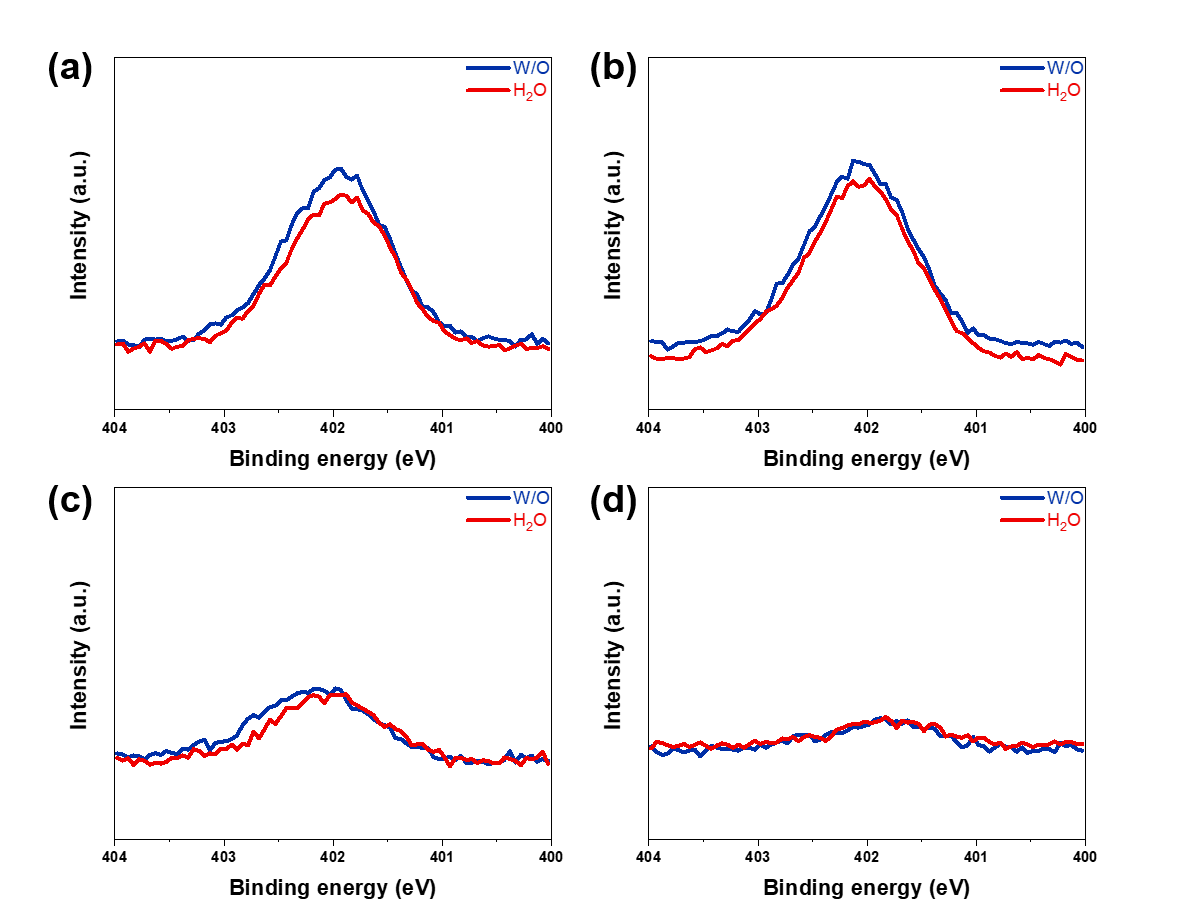


**Fig. S5.** N 1s core-level XPS spectra of the W/O and H_2_O films after annealing for different duration (a) 10 s, (b) 20 s, (c) 40 s and (d) 60 s.


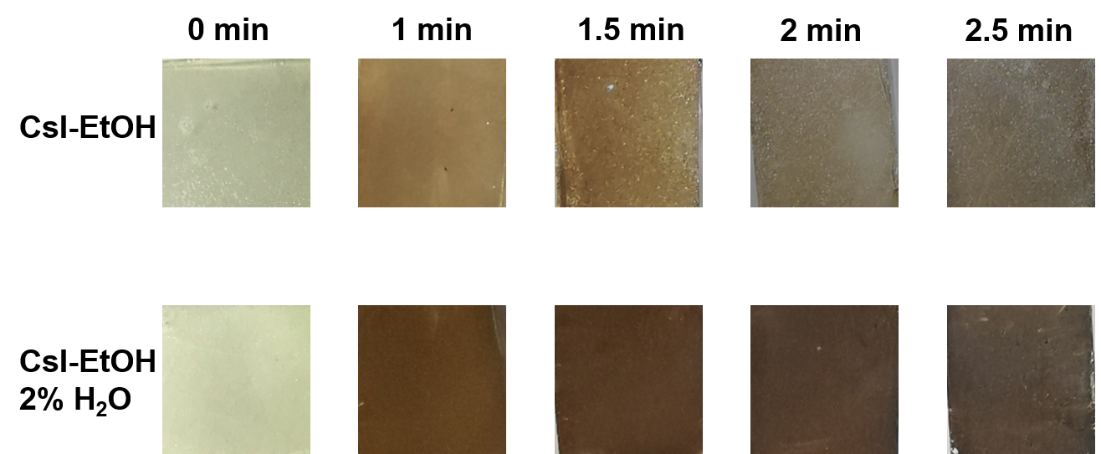


**Fig. S6.** Photographs of the DMAPbI_3_ films after immersion in CsI-EtOH and CsI-EtOH/2% H_2_O for different duration.


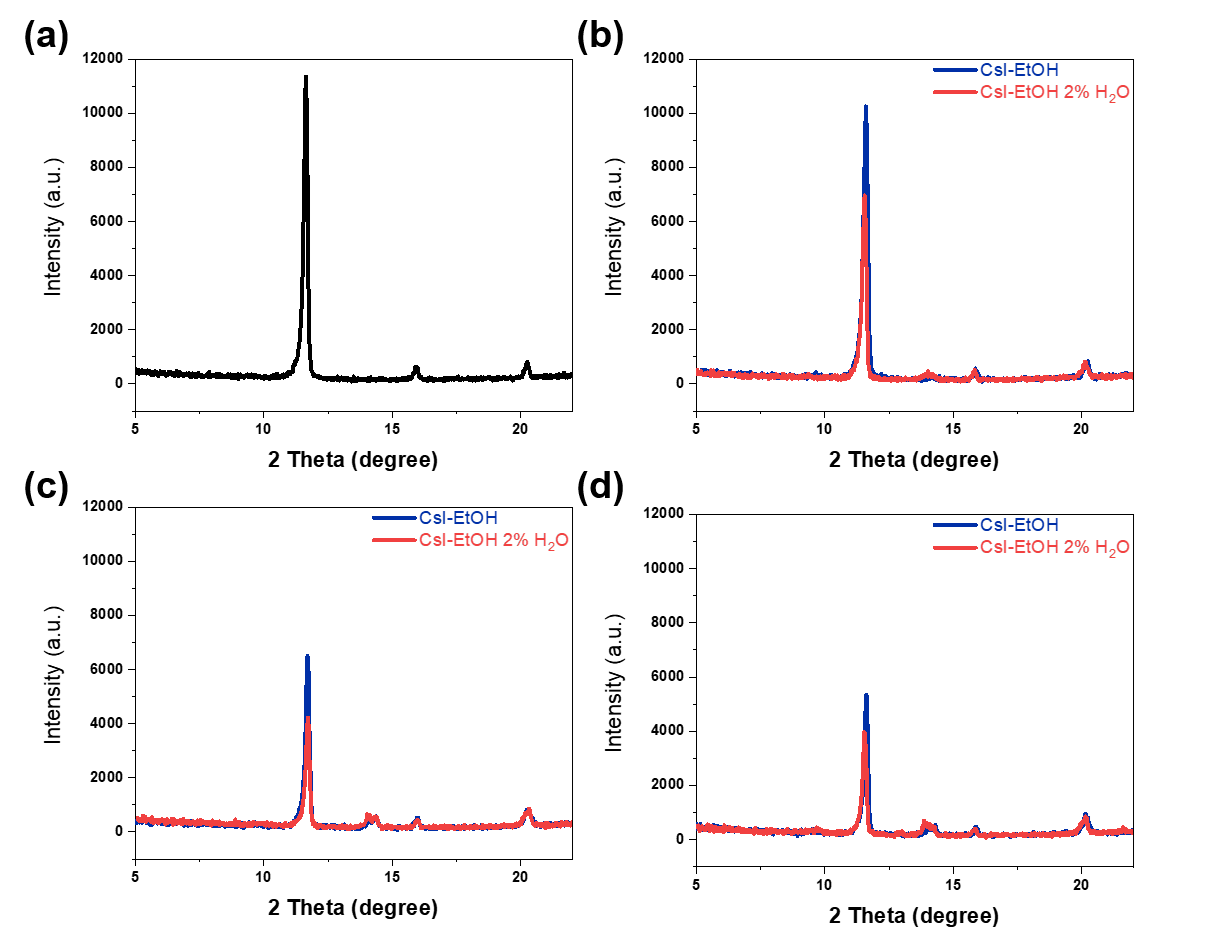


**Fig. S7.** XRD patterns of the DMAPbI_3_ films after soaking in CsI-EtOH and CsI-EtOH/2%H_2_O for different duration: (a) 0 min, (b) 1 min, (c) 1.5 min and (d) 2 min.


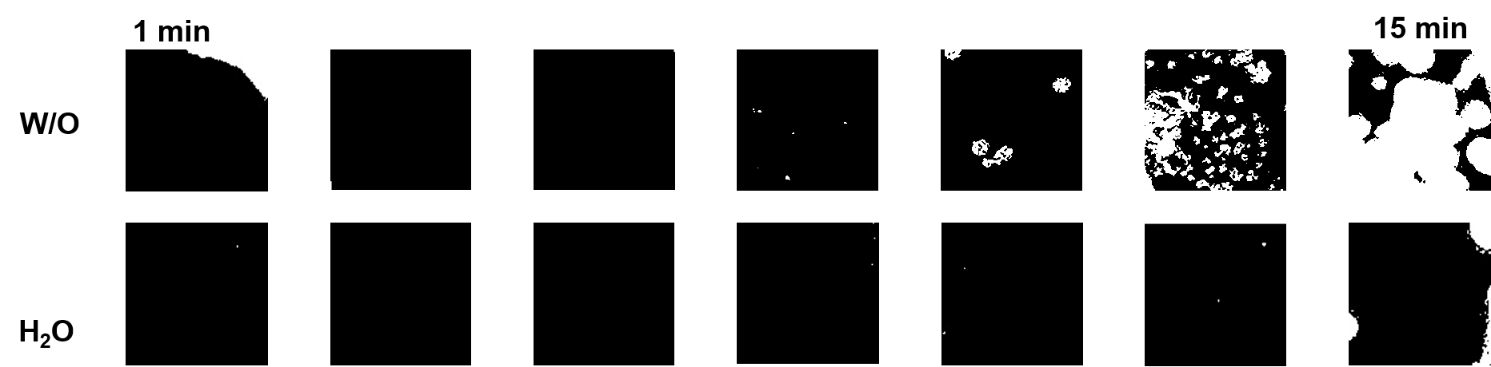


**Fig. S8.** The corresponding binary images obtained from the optical microscope images (Fig. 4(a)).


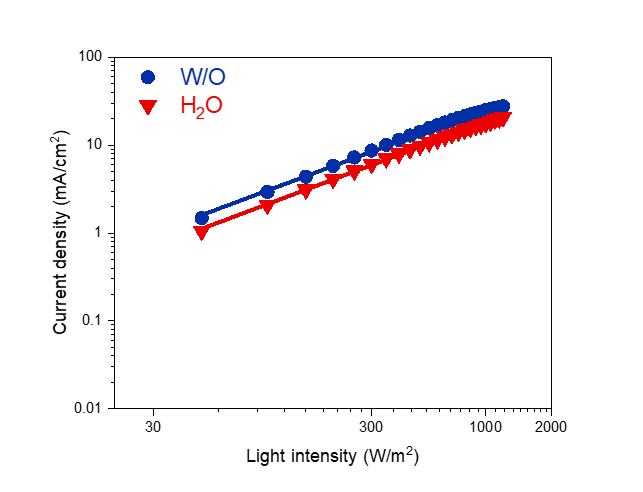


**Fig. S9.** Dependence of J_SC_ on *I* for the W/O and H_2_O C-PSCs.


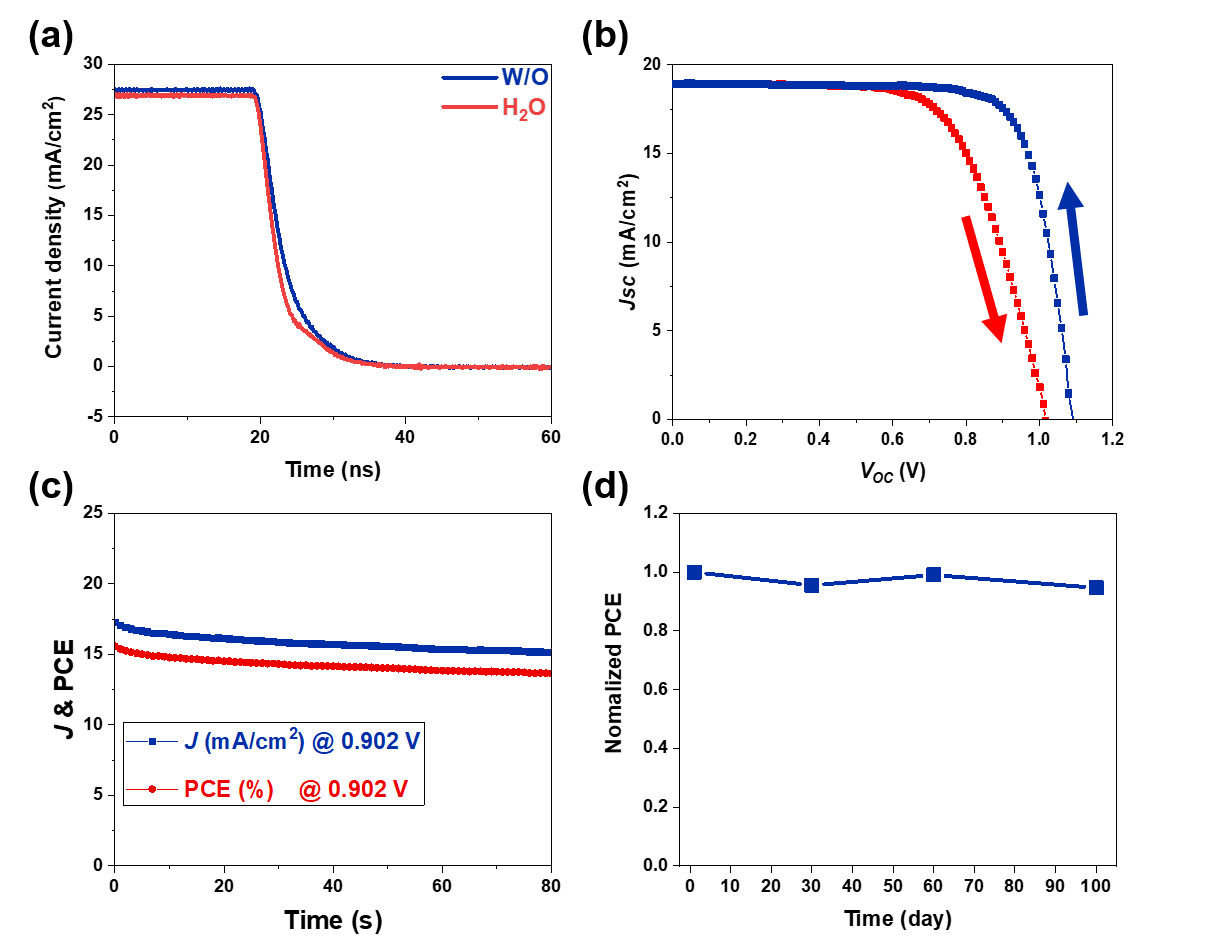


**Fig. S10.** Other photovoltaic performance of C-PSCs. (a) TPC curves, (b) *J-V* curves under forward and reverse scans, (c) steady-state power output, and (d) variation of the normalized PCE with time in dry air (temperature: 20-30 °C; humidity: 10-20%).
